# Whole genome sequence analysis identifies a PAX2 mutation to establish a correct diagnosis for a syndromic form of hyperuricemia

**DOI:** 10.1002/ajmg.a.61814

**Published:** 2020-08-09

**Authors:** Mark Stevenson, Alistair T. Pagnamenta, Silvia Reichart, Charlotte Philpott, Kate E. Lines, OxClinWGS, Caroline M. Gorvin, Karl Lhotta, Jenny C. Taylor, Rajesh V. Thakker

**Affiliations:** 1 Oxford Centre for Diabetes, Endocrinology & Metabolism (OCDEM), Churchill Hospital, University of Oxford, Oxford, UK; 2 Oxford BRC, WCHG, University of Oxford, Oxford, UK; 3 Department of Ophthalmology, Academic Teaching Hospital, Feldkirch, Austria; 4 Department of Internal Medicine III (Nephrology and Dialysis), Academic Teaching Hospital, Feldkirch, Austria

**Keywords:** ADTKD, CKD, optic disc pits, papillorenal syndrome, RCS

## Abstract

Hereditary hyperuricemia may occur as part of a syndromic disorder or as an isolated nonsyndromic disease, and over 20 causative genes have been identified. Here, we report the use of whole genome sequencing (WGS) to establish a diagnosis in a family in which individuals were affected with gout, hyperuricemia associated with reduced fractional excretion of uric acid, chronic kidney disease (CKD), and secondary hyperparathyroidism, that are consistent with familial juvenile hyperuricemic nephropathy (FJHN). However, single gene testing had not detected mutations in the uromodulin (*UMOD*) or renin (REN) genes, which cause approximately 30-90% of FJHN. WGS was therefore undertaken, and this identified a heterozygous c.226G>C (p.Gly76Arg) missense variant in the paired box gene 2 (*PAX2*) gene, which co-segregated with renal tubulopathy in the family. PAX2 mutations are associated with renal coloboma syndrome (RCS), which is characterized by abnormalities in renal structure and function, and anomalies of the optic nerve. Ophthalmological examination in two adult brothers affected with hyperuricemia, gout, and CKD revealed the presence of optic disc pits, consistent with optic nerve coloboma, thereby revising the diagnosis from FJHN to RCS. Thus, our results demonstrate the utility of WGS analysis in establishing the correct diagnosis in disorders with multiple etiologies.

## 1 Introduction

Hyperuricemia, which may lead to gout, occurs as an acquired or inherited metabolic abnormality. Acquired hyperuricemia may be due to: a diet high in purines (e.g., meats, fructose, and beer); drugs (e.g., thiazide diuretics, cytotoxic agents, and low dose aspirin); obesity and metabolic syndrome as a consequence of insulin resistance and the role of insulin reducing urinary urate excretion; hypertension resulting in renal vasoconstriction and uric acid retention; chronic kidney disease (CKD) and renal failure; and low level lead and cadmium intoxication (Choi, Atkinson, Karlson, Willett, & Curhan, 2004; Johnson et al., 2013; Lin, Ho, & Yu, 1999; Messerli, Frohlich, Dreslinski, Suarez, & Aristimuno, 1980; Quinones Galvan et al., 1995; Sharon & Schlesinger, 2016). Hereditary hyperuricemia may occur as an isolated nonsyndromic disease or as part of a syndromic disorder (Megaw, Lampe, Dhillon, Yoshida, & Wright, 2013; Partington & Hennen, 1967; Sperling, Sarapers, Eilam, & Devries, 1972). Genome wide association studies (GWAS) have reported associations between hyperuricemia and approximately 30 loci (e.g., *GLUT9*, *SLC2A9*, *ABCG2*, *SLC17A3*, *SLC17A1*, *SLC22A11*, *SLC22A12*, *GCKR*, *LRRC16A*, *PDZK1*, *R3HDM2-INHBC*, *RREB1*, *TRIM46*, *INHBB*, *SFMBT1*, *TMEM171*, *VEGFA*, *BAZ1B*, *PRKAG2*, *STC1*, *HNF4G*, *A1CF*, *ATXN2*, *UBE2Q2*, *IGF1R*, *NFAT5*, *MAF*, *HLF*, *ACVR1B-ACVRL1*, and *B3GNT4*) (Dehghan et al., 2008; Doring et al., 2008; Kolz et al., 2009; Kottgen et al., 2013; Li et al., 2007; Vitart et al., 2008; Yang et al., 2010), and approximately 20 syndromes associated with hyperuricemia are listed on the Online Mendelial Inheritance in Man (OMIM) database, and these include the Lesch-Nyhan syndrome (MIM 300322), phosphoribosylpyrophosphate synthetase superactivity (MIM 300661), medullary cystic kidney disease (MCKD; MIM 603860), and familial juvenile hyperuricemic nephropathy (FJHN; MIM 162000).

FJHN, which is a genetically heterogeneous disorder, is characterized by hyperuricemia, reduced fractional excretion of uric acid (FEUA), gout, and progressive end stage renal disease (ESRD) associated with interstitial fibrosis. FJHN in approximately 25-85%, <5%, <1%, and <5% of patients is associated with mutations of the *UMOD*, renin (*REN*), protein transport protein SEC61 translocon subunit alpha 1 (SEC61A1), and hepatocyte nuclear factor 1 homeobox B (*HNF-1β*) genes, respectively (Bleyer, Kidd, Zivna, & Kmoch, 2017; Clissold, Hamilton, Hattersley, Ellard, & Bingham, 2015; Dahan et al., 2003; Devuyst et al., 2019; Devuyst, Olinger, & Rampoldi, 2017; Kudo et al., 2004; Piret et al., 2011; Simmonds, Cameron, Goldsmith, Fairbanks, & Raman, 2006; Stacey et al., 2003; Stiburkova, Majewski, & Hodanova, 2002; van der Made et al., 2015; Venkat-Raman, Gast, Marinaki, & Fairbanks, 2016; Vylet’al et al., 2006; Williams et al., 2009). A further FJHN locus has been mapped to chromosome 2p22.1-2p21.2, but its causative gene defect has yet to be identified (Piret et al., 2011).

Here, we report a kindred considered to have FJHN on the basis of hyperuricemia, gout, reduced FEUA, and CKD, but in whom Sanger DNA sequence analysis had not detected mutations of *UMOD* or *REN*, which account for approximately 30-90% of cases. However, whole genome sequence (WGS) analysis unexpectedly revealed that a mutation of the paired box 2 (PAX2) gene was the likely cause of FJHN in this kindred, which prompted clinical reassessment of the family.

## 2 Materials and Methods

### 2.1 Editorial policies and ethical considerations

Informed consent and venous blood samples were obtained from nine available members (comprising five affected and four unaffected members) of the family with suspected FJHN, using protocols approved by the Multicentre Research Ethics Committee (UK) (MREC/02/2/93), and local ethics committees (Austria).

### 2.2 Patients and clinical findings

**Figure 1:**
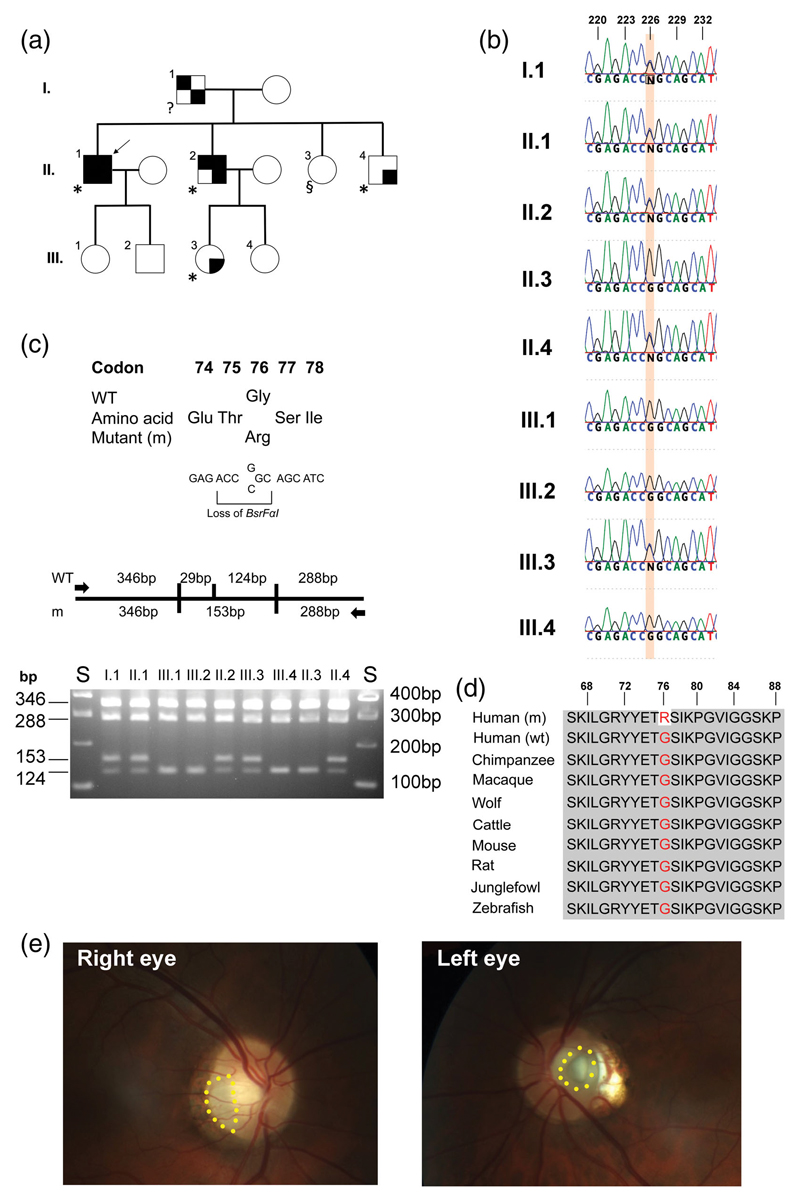
(a) Pedigree of affected proband (individual II.1, indicated with an arrow), with four affected relatives (individuals I.1, II.2, II.4, and III.3) and four unaffected relatives (individuals II.3, III.1, III.2, and III.4). Males: square; females: circle. Open symbols: unaffected; filled top left quadrant: kidney disease; filled top right quadrant: hyperuricemia; filled bottom left quadrant: secondary hyperparathyroidism; and filled bottom right quadrant: proteinuria and/or albuminuria. *optic nerve pathology; ^§^no optic nerve pathology; ^?^optic nerve pathology status unknown. (b) DNA sequence analysis showing c.226G>C (highlighted) within exon 3 of *PAX2*. The DNA sequence chromatograms show that the affected proband (individual II.1), his affected father (individual I.1), affected brothers (individuals II.2 and II.4), and affected niece (individual III.3), are heterozygous G/C, while the unaffected relatives (individuals II.3, III.1, III.2, and III.4) are all homozygous G/G. (c) The *PAX2* c.226G>C mutation is predicted to lead to a missense substitution of Gly, encoded by GGC, to Arg, encoded by CGC, at codon 76 and result in the loss of a *BsrFαI* RE site (R/CCGG/Y). Restriction maps show that the *BsrFαI* digest would result in four products for the wild-type (WT), and three products for the mutant (m). RE digest of *PAX2* exon 3 PCR products demonstrating that the affected individuals I.1, II.1, II.2, II.4, and III.3 are heterozygous for WT (346, 288,124, and 29 bp [not shown]), and m (346, 288, and 153 bp) alleles, and unaffected relatives II.3, III.1, III.2, and III.4 are homozygous for WT alleles. S, size marker. (d) Multiple protein sequence alignment of *PAX2* residues comprising a paired domain involved in DNA binding. Conserved residues are shown in gray, and wild-type Gly76 (G76) and mutant Arg76 (R76) are shown in red. (e) Ophthalmological examination of proband II.1 showing dysplastic optic nerve (indicated by a dotted yellow line) in the right eye and an optic disc pit (indicated by a dotted yellow line) in the left eye

The proband (Figure 1a, individual II.1), a 57-year-old man, presented with hyperuricemia with reduced FEUA at 32 years of age, and later developed CKD and secondary hyperparathyroidism (Table 1), consistent with FJHN. Histological analysis of a single glomerulus from a kidney biopsy taken at the age of 32 years was suggestive of glomerulonephritis but was considered inconclusive as other glomerula were not present among the biopsy sections to confirm this finding. Electron microscopy of the single glomerulus showed that it was abnormal with segmental lobe collapse, basal membrane ruptures, and segmental sclerosis with numerous tubular-reticular structures. At 53 years of age he had an elevated serum creatinine of 4.2 mg/dl [normal range (NR) = 0.5-1.2 mg/dl], proteinuria of 2,500 mg/g creatinine (NR <110 mg/g), albuminuria of 1,655 mg/g creatinine (NR <3 mg/g), and a reduced FEUA of 4.5% (NR = 7.5 ± 1.8%). He was treated with ramipril 5 mg/day, calcitriol 0.25 μg/day, cholecalciferol 12,000 IU/week, allopurinol 100 mg/day, and bicarbonate 2,500 mg/day. Two years later peritoneal dialysis was started due to end-stage kidney disease. The proband’s brother (individual II.2) was also affected, and presented at the age of 44 years with gout. Clinical evaluation revealed: renal insufficiency with elevated serum creatinine of 1.8 mg/dl; recurrent attacks of gout, hyperuricemia and a reduced FEUA of 4.7%; and proteinuria and albuminuria of 740 and 323 mg/g creatinine, respectively (Table 1). He was treated with ramipril 5 mg and allopurinol 150 mg/day. The proband’s father (individual I.1) had chronic renal failure, with serum creatinine of 1.3 mg/dl, and proteinuria of 1,000 mg/g creatinine (Table 1). The proband’s younger brother (individual II.4) had mild albuminuria of 34 mg/g creatinine, and his niece (individual III.3) had albuminuria of 689 mg/g creatinine and proteinuria of 910 mg/g creatinine (Table 1). The albuminuria observed in patients II.1, II.2, and III.3 was considerably higher than that reported previously in other patients with FJHN (Eckardt et al., 2015; Lee, Kim, Oh, Noh, & Lee, 2010). Mutational analysis of the *UMOD* and *REN* genes using leukocyte DNA from the proband did not detect any abnormalities.

### 2.3 WGS and variant confirmation

Leukocyte DNA was used for WGS (Supporting Information Methods), utilizing DNA from two affected individuals [individuals II.1 and II.2 (Table 1 and Figure 1)]. Variants were confirmed by DNA Sanger sequence analysis using PCR products that were generated using *PAX2* forward (5’-AGT AGG AAA GGG CTC GAG GTG GT-3’) and reverse (5’-GGA GAA GCC TGG CAG GGA ATA-3’) primers (Life Technologies), the BigDye Terminator v3.1 Cycle Sequencing Kit (Life Technologies) and an automated detection system (ABI3730 Automated capillary sequencer; Applied Biosystems). Further validation was performed by *BsrFαI* (New England Biolabs) restriction endonuclease (RE) digestion of PCR products according to the manufacturer’s guidelines.

## 3 Results

WGS analysis of leukocyte DNA from two affected individuals (Figure 1a, II.1 and II.2) confirmed the absence of *UMOD* and *REN* abnormalities, and also an absence of abnormalities within the *SEC61A1* and *HNF-1β* genes that have been reported to be associated with FJHN. Futhermore, copy number variants (CNVs) were not identified in these four genes, and an examination of all rare (allele frequency <3%) variants in these genes also did not reveal any deleterious alleles to be shared by the two affected brothers, II.1 and II.2 (Table S1). CNVs in three other genes (*LINC01060, NRG3*, and *PMM2*) were found (Table S2), but were not further investigated as they were highly unlikely to be causative of the phenotypic abnormalities. However, WGS analysis identified a heterozygous G-to-C transversion at nucleotide c.226 in exon 3 of *PAX2* (NM_003987.3) that was confirmed by DNA Sanger sequence analysis (Figure 1b). This G-to-C transversion (GGC to CGC), which predicts a missense substitution (p.Gly76Arg) of the PAX2 protein led to the loss of a *BsrFaI* RE site (Figure 1c). Analysis of the nine available family members (5 affected and 4 unaffected members) by DNA Sanger sequencing (Figure 1b) and RE digestion (Figure 1c) revealed co-segregation of the c.226G>C variant and FJHN phenotype. Thus, the heterozygous *PAX2* c.226G>C variant was present in the five affected individuals (I.1, II.1, II.2, II.4, and III.3), but not in the four unaffected individuals (II.3, III.1, III.2, and III.4) that were homozygous for the wild-type c.226G (Figure 1b,c). Moreover, this *PAX2* c.226G>C variant was absent from the greater than 125,000 exomes and greater than 15,000 genomes contained within the Genome Aggregation Database (gnomAD v2.1.1) database (Karczewski et al., 2020). Analysis of p. Gly76Arg using SIFT (http://sift.jcvi.org/), Mutation Taster (http:www.mutationtaster.org/), and PolyPhen-2 (http://genetics.bwh.harvard.edu/pph2/) predicted the variant to be “Deleterious,” “Disease Causing,” and “Probably Damaging,” respectively. Gly76, which is located in the paired domain of *PAX2*, lies within a stretch of evolutionarily highly conserved residues (Figure 1d), and this further supports the pathogenicity of the p.Gly76Arg variant. In addition, a different missense mutation at this same residue (p.Gly76Ser) has been reported in patients with renal coloboma syndrome (RCS), and these combined observations help support that the p.Gly76Arg identified in this family (Figure 1a-c) is also a disease-causing variant. RCS, which is also known as papillorenal syndrome (PAPRS) (MIM 120330) (Devriendt et al., 1998), is characterized by renal and ocular anomalies that include renal hypodysplasia and insufficiency progressing to ESRD, and optic nerve coloboma. RCS has been reported to be associated with hyperuricemia and gout in two unrelated families (Deng et al., 2019; Megaw et al., 2013) and the finding of the PAX2 Gly76Arg mutation in the family with FJHN (Figure 1a-c) prompted an ophthalmological examination of the proband (II.1). This revealed the presence of a dysplastic papilla with temporal inferior pallor in the right eye and of an optic disc pit in the left eye (Figure 1e), consistent with optic nerve coloboma in both eyes. Subsequent ophthalmological examinations of the affected brothers (II.2 and II.4) and niece (III.3) revealed the presence of unilateral optic nerve colobomas only, in all of them. Other ocular abnormalities were not identified in any of these four affected individuals (II.1, II.2, II.4, and III.3), and ophthalmological examination of the unaffected sister (II.3) also revealed no abnormalities. The findings in the four affected individuals (II.1, II.2, II.4, and III.3) are consistent with a diagnosis of RCS, which has been reported to be also associated with anomalies of the central nervous system (CNS), intellectual disability, hearing loss, joint laxity, and elevations of pancreatic amylase; and these individuals were therefore further assessed for such manifestations. This revealed that none of the individuals had: clinical signs of CNS anomalies, and magnetic resonance imaging (MRI) of the brain in individuals II.1 and II.2, has revealed the occurrence of only of an empty sella turcica in individual II.1; intellectual disability; hearing loss, except individual II.4 who is reported to have mild hearing loss but has declined formal hearing tests; joint laxity; a history of pancreatitis; or elevated pancreatic amylase, which has been assessed in only individual II.1.

## 4 Discussion

Our study reports a kindred affected with CKD, reduced FEUA, hyperuricemia, and gout, which were consistent with a diagnosis of FJHN. However, the kindred did not have *UMOD*, *REN*, *SEC61A1*, or *HNF-1β* gene mutations, which collectively are associated with approximately 30-90% of FJHN cases, but instead had a missense mutation (p.Gly76Arg) of PAX2, whose abnormalities are more commonly associated with RCS. Indeed, ophthalmic examination, prompted after the identification of the *PAX2* mutation by WGS, identified optic nerve abnormalities consistent with RCS, in all four affected family members that were available for ophthalmic assessments (Figure 1a-e).

RCS is characterized by abnormalities in renal structure and function in greater than 90% of patients, ophthalmological anomalies in greater than 75% of patients, and hearing loss in less than 10% of patients (Bower et al., 2012). The most common renal findings are renal hypodysplasia, vesicoureteral reflux (VUR), renal cysts, and multicystic dysplastic kidneys, which occur in 65%, ~15%, <10%, and ~5% of patients, respectively. Renal failure is reported in approximately 15% of cases, while CKD stage 5 requiring a kidney transplant is common and has a range of onset from birth to greater than 75 years of age (Bower et al., 2012). The ophthalmoscopic findings include optic nerve coloboma, optic disc dysplasia, excavation of the optic disc or optic disc “pits,” morning glory anomaly, and hypoplastic optic discs, which occur in ~50%, >10%, <10%, ~5%, and <5% of patients, respectively (Bower et al., 2012). Retinal, macular, and lens abnormalities have also been reported in some patients (Bower et al., 2012). PAX2 is expressed in other tissues (e.g., cerebellum, hypothalamus otic vesicle, genitourinary tract, and pancreas), and additional features of RCS include CNS anomalies, intellectual disability and elevated pancreatic amylase (Bower et al., 2012).

A frameshift deletion of *PAX2* in a family with optic nerve colobomas, renal hypoplasia and VUR (Sanyanusin et al., 1995) represents the first reported single gene defect causation of congenital anomalies of the kidney and urinary tract (CAKUT). Subsequently, larger patient cohort studies confirmed *PAX2* mutations as an important cause of syndromic CAKUT and the establishment of RCS as a separate disease entity (Madariaga et al., 2013; Rossanti et al., 2020; Thomas et al., 2011; Weber et al., 2006). *PAX2* is a member of the paired box (PAX) family of transcriptional regulatory genes with nine members described in humans. The majority of *PAX2* pathogenic mutations are located in the paired domain (comprising a conserved 128 amino acid region) that has DNA binding properties encoded by exons 2-4 (Bower et al., 2012; Eccles et al., 2002). However, evidence from an international consortium of three laboratories collecting data on *PAX2* mutations in RCS patients reported that there are no clear genotype/phenotype correlations, and variable types of *PAX2* mutation (missense, frameshifts, splice sites, and deletions) located across 10 of the 12 *PAX2* exons can lead to similar phenotypes, while the same mutation within members of the same family can have variable penetrance and manifestations of RCS (Bower et al., 2012). This large intrafamily variability in RCS suggests that factors other than PAX2 may play a role in clinical penetrance (Bower et al., 2012). *PAX2* mutations are found in approximately 50% of RCS/PAPRS, thereby suggesting that other abnormalities of genes may be involved in the etiology of this disorder (Dureau et al., 2001; Okumura et al., 2015).

The presence of optic disc pits and dysplastic papilla in the family reported here (Figure 1a,e) is a distinguishing feature confirming RCS from FJHN given that CKD is common to both. This family also has reduced FEUA, hyperuricemia and gout that are commonly found in FJHN. Such occurrence of RCS with hyperuricemia and gout, has been previously reported in only two unrelated families (Megaw et al., 2013). One family, which had a *PAX2* frameshift mutation [c.567_568dup (p. Ile190ArgfsX85)] in exon 5, consisted of five affected males from three generations; all the five affected males suffered from hyperuricemia and/or gout and the proband also suffered from diabetes mellitus and cryptorchidism, which have not previously been associated with RCS (Megaw et al., 2013). In the other family, a de novo heterozygous C-to-T transition (c.418C>T) in exon 4 of *PAX2* that would result in a missense Arg140Trp mutation was identified in a 14.8 year old girl who presented with hyperuricemic gout, in association with renal disease and ophthalmic abnormalities consistent with RCS (Deng et al., 2019). These reports together with our findings of a *PAX2* c.226G>C transversion in exon 3 that resulted in a missense Gly76Arg mutation in a kindred with hyper-uricemic nephropathy and features of RCS (Figure 1a-c), suggest that the association of gout with RCS may not be rare.

The identification of a *PAX2* mutation in the family (Figure 1a-c) reported in this study and the subsequent revision of the diagnosis from FJHN to RCS will have important implications for improved patient care, both in terms of treatments for the features already manifested, and also for longer term monitoring of other RCS associated phenotypes that may develop in the future. Thus, the patients and their unaffected relatives in the family (Figure 1a-c) have been informed of the results from the genetic testing, and those having the mutation have been provided with details about the clinical manifestations and management of RCS, which includes: the likelihood of developing kidney failure and the necessity of having regular hospital appointments for assessments of renal function; the mode of inheritance; and the risks for their children inheriting the mutation and developing RCS. Equally important, the confirmation of the absence of the mutation in the unaffected family members (II.3, III.1, III.2, and III.4) will also alleviate concerns for these individuals over nonpenetrance of the disease and reduce the burden of monitoring since they are at greatly reduced risk of developing kidney disease.

WGS, which enables the detection of all classes of genetic change including SNV, CNV, translocations, and variants in noncoding regions that may confer a pathogenic effect, has become increasingly affordable in recent years and provides a method to aid diagnosis of complex diseases with genetic etiologies. Thus, we have demonstrated that WGS can improve diagnosis of inherited forms of hyperuricemia and kidney disease, which can be challenging to achieve by pathological and biochemical analysis alone.

## Supplementary Material

Online Supplementary Methods

Supplementary Table 1

Supplementary Table 2

## Figures and Tables

**Table 1:** Clinical details of affected and unaffected members of the kindred with chronic kidney disease (CKD)

	Individual								
	I.1	II.1	II.2	II.3	II.4	III.1	III.2	III.3	III.4
Chronic kidney disease^a^	G3aA3	G5D	G3bA3	−	G2A2	−	−	G2A3	−
Serum creatinine (mg/dl) (NR 0.5-1.2 mg/dl)	1.3	4.2	1.8	*0.83*	1.05	*0.63*	*1.05*	0.92	*0.86*
Estimated glomerular filtration rate (NR >90 ml/min/1.73 m^2^)	48	15	44	*80*	84	*122*	*97*	86	*95*
Proteinuria (mg/g creatinine) (NR <110 mg/g)	1,000	2,500	740	−	100	−	−	910	−
Albuminuria (mg/g creatinine) (NR <3 mg/g)	−	1,655	323	*<3*	34	*<3*	*<3*	689	*<3*
Secondary hyperparathyroidism	−	+	−	−	−	−	−	−	−
Hyperuricemia	−	+	+	−	−	−	−	−	−
Gout	−	+	+	−	−	−	−	−	−
FEUA (%) (NR 7.5 ±1.8%)	−	4.5	4.7	−	7.7	−	−	−	−
PAX2 mutation (p.Gly76Arg)	+	+	+	−	+	−	−	+	−
Ocular abnormality	NT	Bilateral	Unilateral	−	Unilateral	*NT*	*NT*	Unilateral	*NT*
Current age	93	57	53	*56*	30	*31*	*29*	29	*27*
Age of onset	Unknown^b^	32 (gout)	44(gout)	−	−^c,d^	−	−	−^c^	−

*Note:* + = present; − = absent/not reported; NT = not tested. Individuals II.3, III.1, III.2, and III.4, who had normal renal function and absence of the PAX2 p.Gly76Arg mutation and are unaffected, are shown in italics, while individuals that are not in italics are affected. Estimated glomerular filtration rate was calculated using the chronic kidney disease epidemiology collaboration (CKD-EPI) formula. Abbreviation: FEUA, fractional excretion of uric acid.

a CKD stages according to the Kidney Disease: Improving Global Outcomes (KDIGO) classification (Kidney International Supplements Volume 3, Issue 12,013, KDIGO 2012 Clinical Practice Guideline for the Evaluation and Management of Chronic Kidney Disease).

b Suffers from dementia so age of onset unknown.

c Asymptomatic mutation carrier.

d Mild hearing loss reported.

## Data Availability

The data that support the findings of this study are available from the corresponding author on reasonable request.
