# Supplementary material for: Whole genome sequence analysis identifies a PAX2 mutation to establish a correct diagnosis for a syndromic form of hyperuricemia
Source: Am J Med Genet A. Author manuscript; Available in PMC 2021 Jun 21. (PMC7611017; doi:10.1002/ajmg.a.61814)
Supplement: Online Supplementary Methods [file EMS127174-supplement-Online_Supplementary_Methods.docx]

**ONLINE SUPPLEMENTARY METHODS**

**Genome sequencing and data analysis**

Leukocyte DNA was extracted from venous blood using the Gentra Puregene blood kit (Qiagen) and standard protocols, quantified using a Nanodrop spectrophotometer, and assessed for integrity using an agarose gel, as described [Nesbit et al. 2013]. Genome sequencing was performed in the two affected brothers by Oxford Molecular Diagnostic Centre at the Department of Oncology. Bioinformatic analysis was performed at the Wellcome Centre for Human Genetics (WCHG) in Oxford. The genomes were sequenced to mean coverage of >50x. Paired end 151bp Illumina reads were mapped to hs37d5 using Stampy (version 1.0.23). After removal of duplicate reads using picard (version 1.111), variants were called with Platypus (version 0.8.1) with minFlank option set to 0. Variants were filtered with Ingenuity Variant Analysis software ([www.qiagenbioinformatics.com/products/ingenuity-variant-analysis/](http://www.qiagenbioinformatics.com/products/ingenuity-variant-analysis/)).  As well as passing several confidence filters, variants were required to: 1) have a population allele frequency of <0.1% in 1000 genomes project (phase3v5b), NHLBI ESP exomes (All), ExAC (v0.3.1, Maximum Frequency) and gnomAD (v. 2.0.1, Maximum Frequency); 2) be protein altering (i.e. frameshift, in-frame indel, stop codon change, missense; or disrupt splice site up to 2bp into intron; or predicted to disrupt splicing by MaxEntScan; and 3) be called as heterozygous in both affected brothers. Copy number variations (exonic deletions and duplications) were scrutinized as described [Schuh et al. 2018].

Nesbit MA, Hannan FM, Howles SA, Reed AA, Cranston T, Thakker CE, Gregory L, Rimmer AJ, Rust N, Graham U, Morrison PJ, Hunter SJ, Whyte MP, McVean G, Buck D, Thakker RV. 2013. Mutations in AP2S1 cause familial hypocalciuric hypercalcemia type 3. Nat Genet 45:93-97. 10.1038/ng.2492

Schuh A, Dreau H, Knight SJL, Ridout K, Mizani T, Vavoulis D, Colling R, Antoniou P, Kvikstad EM, Pentony MM, Hamblin A, Protheroe A, Parton M, Shah KA, Orosz Z, Athanasou N, Hassan B, Flanagan AM, Ahmed A, Winter S, Harris A, Tomlinson I, Popitsch N, Church D, Taylor JC. 2018. Clinically actionable mutation profiles in patients with cancer identified by whole-genome sequencing. Cold Spring Harb Mol Case Stud 4:10.1101/mcs.a002279
