# Supplementary material for: Whole genome sequence analysis identifies a PAX2 mutation to establish a correct diagnosis for a syndromic form of hyperuricemia
Source: Am J Med Genet A. Author manuscript; Available in PMC 2021 Jun 21. (PMC7611017; doi:10.1002/ajmg.a.61814)
Supplement: Supplementary Table 1 [file EMS127174-supplement-Supplementary_Table_1.docx]

| Gene | Variant | Region | Transcript ID | Reason for Exclusion |
| --- | --- | --- | --- | --- |
| *UMOD* | None found |  |  |  |
| *REN* | None found |  |  |  |
| *SEC61A1* | c.-450C>G | Promoter | NM_013336.4 | Not present in both brothers |
| *SEC61A1* | c.616+233G>A | Intronic | NM_013336.4 | Not present in both brothers |
| *HNF1B* | c.1046-3158T>A | Intronic | NM_000458.4 | Not present in both brothers |
| *HNF1B* | c.1046-9464C>T | Intronic | NM_000458.4 | Not present in both brothers |
| *HNF1B* | c.1045+6684_1045+6687dupACAC | Intronic | NM_000458.4 | Not present in both brothers |
| *HNF1B* | c.1045+5433_1045+5434delGA | Intronic | NM_000458.4 | Not present in both brothers |
| *HNF1B* | c.1045+5433_1045+5434dupGA | Intronic | NM_000458.4 | Not present in both brothers |
| *HNF1B* | c.545-2701A>C | Intronic | NM_000458.4 | Not present in both brothers |

Supplementary Table 1. List of rare genomic variants in the four genes associated with FJHN identified by WGS. Following a prefilter to exclude common variants (allele frequency >3%) a total of 322,018 genomic variants were identified of which 301,879 passed quality call control and read depth filters, and excluded the 1% most exonically variable genes in healthy public genomes.
