# Supplementary material for: Whole genome sequence analysis identifies a PAX2 mutation to establish a correct diagnosis for a syndromic form of hyperuricemia
Source: Am J Med Genet A. Author manuscript; Available in PMC 2021 Jun 21. (PMC7611017; doi:10.1002/ajmg.a.61814)
Supplement: Supplementary Table 2 [file EMS127174-supplement-Supplementary_Table_2.docx]

| Gene | Copy Number Event | Region | Type | Length | Reason for Exclusion |
| --- | --- | --- | --- | --- | --- |
| *LINC01060* | Loss | chr4:189,516,918-189,524,967 | Exonic | 8050 | Variants within the *LINC01060* gene are reported to be associated with 12 phenotypes in the National Human Genome Research Institute – European Bioinformatics Institute (NHGRI-EBI) human genome wide association studies (GWAS) catalogue*, none of which were observed in this family. In combination, both brothers also have 12 heterozygous intronic *LINC01060* variants (10SNVs, 1 insertion and 1 deletion) that are not predicted to affect splicing or the expression of the long intergenic non-protein coding RNA 1060. |
| *NRG3* | Loss | chr10:83,941,041-83,960,842 | Intronic | 19802 | Variants within the *NRG3* gene are reported to be associated with 23 phenotypes in the NHRGI-EBI GWAS catalogue, which includes type 1 diabetes nephropathy and resulting chronic kidney disease, however, the intronic loss within the *NRG3* gene is not predicted to affect splicing or the expression of the neuroregulin-3 protein. In combination, both brothers also have 34 heterozygous intronic *NRG3* variants (21 SNVs, 8 insertions and 5 deletions) and 2 homozygous intronic *NRG3* variants (1 insertion and 1 deletion), with a further 6 *NRG3* variants that were homozygous in one brother and heterozygous in the other (1 SNV, 2 insertions and 3 deletions), none of which are predicted to affect splicing or the expression of neuroregulin-3. Optic colobomas are not reported to be a feature of *NRG3* mutations. |
| *PMM2* | Loss | chr16:8,891,875-8,925,501 | Exonic | 33627 | Variants within the *PMM2* gene are reported to be associated with 2 phenotypes in the NHRGI-EBI GWAS catalogue, neither of which were observed in this family. In combination, both brothers also have an intronic heterozygous *PMM2* variant (SNV) that is not predicted to affect splicing or the expression of the phosphomannomutase 2 protein. Optic colobomas are not a feature of *PMM2* mutations. |

Supplementary Table 2. Details of three genes identified with Copy Number Variants (CNVs) in both affected brothers (II.1 and II.2, Figure 1). CNVs were not identified in *UMOD*, *REN*, *SEC61A1* or *HNF-1β* genes.

*Buniello A, MacArthur JAL, Cerezo M, Harris LW, Hayhurst J, Malangone C, McMahon A, Morales J, Mountjoy E, Sollis E, Suveges D, Vrousgou O, Whetzel PL, Amode R, Guillen JA, Riat HS, Trevanion SJ, Hall P, Junkins H, Flicek P, Burdett T, Hindorff LA, Cunningham F and Parkinson H.
The NHGRI-EBI GWAS Catalog of published genome-wide association studies, targeted arrays and summary statistics 2019.
Nucleic Acids Research, 2019, Vol. 47 (Database issue): D1005-D1012.
